# Semen cryopreservation for the Mediterranean brown trout of the Biferno River (Molise-Italy): comparative study on the effects of basic extenders and cryoprotectants

**DOI:** 10.1038/s41598-019-45006-4

**Published:** 2019-07-04

**Authors:** Michele Di Iorio, Stefano Esposito, Giusy Rusco, Alessandra Roncarati, Marsia Miranda, Pier Paolo Gibertoni, Silvia Cerolini, Nicolaia Iaffaldano

**Affiliations:** 1 Department of Agricultural, Environmental and Food Sciences, University of Molise, Campobasso, Italy; 2 Mediterranean Trout Research Group – Centro di ricerche “I Giardini dell’Acqua”, 42037 Collagna, RE Italy; 3 School of Biosciences and Veterinary Medicine, University of Camerino, Viale Circonvallazione 93-95, Matelica, Italy; 4 Department of Veterinary Medicine, University of Milan, Milan, Italy

**Keywords:** Zoology, Ichthyology

## Abstract

This study was designed to optimize the semen freezing protocol of the native Mediterranean brown trout inhabiting the Molise rivers through two experiments: an *in vitro* analysis of the effects of two basic extenders combined with three cryoprotectants on post-thaw semen quality; and an *in vivo* test to assess the fertilization and hatching rate. Semen was diluted at a ratio of 1:3 in a freezing medium composed of a glucose extender (A) or mineral extender (B). Each basic component contained 10% dimethylsulfoxide, dimethylacetamide or methanol. The post-semen quality was evaluated considering motility, duration of motility, viability and DNA integrity. The basic extender and cryoprotectant were shown to have significant effects on these variables, and the best results were obtained using extender A or B combined with dimethylsulfoxide (P < 0.05). These freezing protocols were selected for fertilization trials *in vivo*. Fertilization and hatching rates were significantly higher in fresh semen. No significant differences were observed in frozen semen using extender A or B, although higher percentages of eyed eggs and hatching rates were recorded using extender A. According to our *in vitro* and *in vivo* results, the glucose-based extender and dimethylsulfoxide emerged as the best combination for an effective cryopreservation protocol for semen of this trout.

## Introduction

Cryobanking is a valuable tool to preserve the genetic resources of a wide range of fish species and cryopreservation has been extensively used in assisted reproductive technology, agriculture, and conservation programmes for endangered species playing an important role in genetic selection programmes, biodiversity conservation and restocking programmes^1–3^.

In aquaculture, semen cryopreservation is widely used to provide gametes year-round for fertilization outside of the normal reproductive seasons or to promote alternative techniques for broodstock management. In the case of some catfish, the study of sperm cryopreservation is more extensive than studied concerning females because the availability male fish is often limited and their peak spawning occurs at different times of the year, thus sperm cryopreservation can be used strategically to improve the production of hybrid catfish^4^. In endangered wild aquatic species, gamete freeze preservation is a valuable tool for preserving the genetic material of native populations with a specific genotype^5^. Research on fish germplasm cryobanking has been carried out on different cell types, including sperm^2^, somatic cells^6^, fish oocytes and embryos^7,8^, and more recently spermatogonia and primordial germ cells^9^.

In this regard, studies carried out so far on the cryopreservation of fish oocytes indicated that some initial promising results were obtained with early stage ovarian follicles, such as stage I and stage II follicles^2^. However, more work needs to be done in optimising the protocols for cryopreservation of fish ovarian follicles. Fish embryo cryopreservation has been a challenging objective for decades and has yet to be achieved. Therefore, successful fish embryo cryopreservation remains elusive^2^. Mainly due to the small size of sperm cells and relatively high resistance to chilling, sperm cryopreservation is more advantageous compared to the one performed on other cell types, thus resulting in this being the most established technique in aquatic species^2^.

Salmonid sperm cryopreservation has been widely studied due to their high commercial value, both in the food sector and for recreational purposes such as fishing^2,10–13^. However, in salmonids many difficulties have been encountered because of the sperm’s high susceptibility to cryopreservation-induced damage caused by the short duration of motility, low ATP production, high sensitivity to osmotic stress and large number of spermatozoa required to fertilize each egg^14^.

Many authors have described damage caused by cryopreservation in trout spermatozoa as affecting motility, cell metabolism, and the structure of the plasma membrane, mitochondria, tail and chromatin^12,15,16^. The plasma membrane is one of the most susceptible structures since it is highly sensitive to cold-shock damage^17^, osmotic stress^18^, and the presence of reactive oxygen species (ROS) generated during the freezing/thawing processes. These agents alter lipid and protein composition, leading to a decrease in sperm quality after cryopreservation^3,19^.

Cryopreservation involves several factors that need to be fine-tuned to improve sperm cryosurvival^13,20^, including the quality of fresh semen, the composition of the basic extender, the type of cryoprotectant (CPA) and its concentration, and the freezing and thawing rate^10–13,21,22^. Among these factors a decisive role is played by the basic extender and CPA type^23^. Two types of basic extenders have generally been used for the cryopreservation of trout spermatozoa: seminal plasma-mimicking media and simple carbohydrate-based solutions^24,25^.

Dimethylsulfoxide (DMSO) is usually applied as the penetrating cryoprotectant (P-CPA) in trout semen; however, other penetrating cryoprotectants (P-CPAs), such as dimethylacetamide (DMA), ethylene glycol, methanol (MeOH), glycerol and DMSO – glycerol mixtures, are also reported to provide efficient results^13,15^.

Listed under the scientific name of *Salmo cettii*^26,27^, the Mediterranean brown trout (*Salmo macrostigma*) inhabiting the Molise rivers is considered “critically endangered” by the Italian Red List of the International Union for Conservation of Nature and Natural Resources (IUCN). Finding an efficient freezing protocol for the semen of the Mediterranean brown trout (*Salmo macrostigma*) of Molise will allow the creation of a sperm cryobank. The sperm cryobank of autochthonous Mediterranean trout populations with high genetic variability represents an action within our financed “Life” project focusing on the recovery and conservation of this native trout in Molise rivers.

Therefore, this study was designed to improve the semen freezing procedure for native Molise trouts for the creation of a sperm cryobank by 1) investigating the effects of two basic extenders combined with three P-CPAs on *in vitro* post-thaw semen quality and 2) assessing the *in vivo* yields of the most effective P-CPA identified during phase 1) for each extender.

## Methods

### Chemicals

The LIVE/DEAD Sperm Viability Kit was purchased from Molecular Probes, Inc. (Eugene, OR, USA) and all other chemicals used in this study were purchased from Sigma, Chemical Co. (Milan, Italy).

### Animal and gamete collection

The experiments were carried out during the spawning season (January-February) in the Bojano spring of the Biferno River (Molise, Italy). This sampling location was selected because it is a highly attractive spawning site used by the native trout population after upstream migration.

A total of 67 autochthonous individuals of *Salmo cettii* were captured by electro-fishing and identified as 60 males and 7 females, based on their phenotypic characteristics^28–30^ and aged as 2+ to 5+ years old. The average total lengths of the fishes were 33.8 ± 5.4 cm for males and 36.5 ± 7.7 cm for females.

Preliminary results of genetic analysis (data not published yet) show that specimens captured in this location have low levels of introgression (approximately 0.15%) among allochthonous brown trouts.

Sperm samples were collected by gentle abdominal massage, and abdomens and urogenital papilla were dried before stripping, with special care to avoid contamination of semen with urine, mucus and blood cells. Each male was stripped once only and the total amount of expressible milt was collected individually in graduated plastic tubes.

The semen was transported from the river to the laboratory in a portable refrigerator at 4 °C. Only spermatozoa showing a motility (subjectively evaluated as described in the sperm function section) rate higher than 75% were used. Ejaculates of different males were pooled (4/5 ejaculates/pool). In total, 12 pools were created and stored in a portable fridge (4 °C) before cryopreservation.

Egg collection from the 7 mature females was also performed through gentle abdominal massage and eggs were carefully selected based on their well-rounded shape and transparency.

The experiments were carried out in accordance with the Code of Ethics of the EU Directive 2010/63/EU for animal experiments. The Bioethics Committee of University of Molise (UNIMOL) approved all procedures performed in this study (protocol n. 450 - UNMLCLE).

This study is part of a Nat.Sal.Mo LIFE project (NAT/IT/000547) financed by the European community. In addition our Life project received “a positive opinion” from the Ministry of the Environment and the Protection of The territory and the Sea. Sampling and handling of fish followed the practices reported in the Ministerial Protocol (ISPRA) in terms of animal welfare.

All experiments were conducted with the appropriate permits of the competent authorities (Molise Region, protocol number 6192, 13/12/2017) according to the current regulations on the protection of the species, bio-security, protocols of sampling of fresh water and animal welfare. Gametes were transported in compliance with current national regulations (Legislative Decree 148/2008, D.L 3/08/2011).

### Experiment 1. Effects of different extenders and cryoprotectants on post-thaw semen quality

The experiment was designed in a 2 × 3 factorial arrangement, in which one of the factors was the extender and the other factor was the cryoprotectant.

#### Extender preparation

Two basic extenders were employed: extender A containing 300 mM glucose^31^ and extender B with 75 mM NaCl, 70 mM KCl, 2 mM CaCl_2_, 1 mM MgSO_4_ and 20 mM TRIS^32^. Each freezing extender contained a basic extender (A or B), +10% DMSO, DMA or MeOH as the P-CPA and 10% of egg yolk as the non-penetrating cryoprotectant (NP-CPA). We utilized the egg yolk because its combined use with P-CPA, for brown trout semen cryopreservation has been reported to be successful^20,25,33^. In total, 6 different freezing extenders (2 basic extenders × 3 penetrating cryoprotectants) were obtained.

#### Sperm cryopreservation

An aliquot taken from each pool was instantly used to assess fresh semen quality as described below. Each pool was split into six equal aliquots (0.4 mL), and each of them was diluted 1:3 (v:v; semen:extender) with the 6 different freezing extenders.

**Figure 1:**
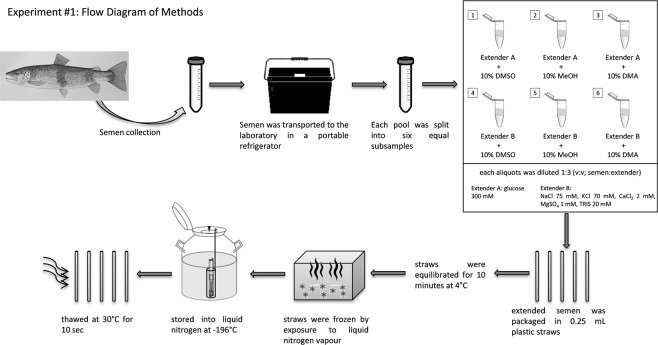
Flow diagram of Experiment #1 demonstrating the cryopreservation protocol used for the brown trout sperm cells as described in Experiment 1.

The extended semen was packaged in 0.25 mL plastic straws (IMV Technologies, L’Agile, France), which were later sealed with polyvinyl alcohol (PVA). In total 360 straws (5 straw for each treatment × 6 treatments × 12 pools) were used. The straws were then equilibrated for 10 minutes at 4 °C (equilibration time), and frozen by exposure to liquid nitrogen vapor at 5 cm above the liquid nitrogen surface for a period of 10 minutes. This method of exposure to liquid nitrogen vapour was previously shown to be appropriate^22^. After cryopreservation, the straws were plunged into liquid nitrogen for storage at −196 °C. Semen samples were later thawed in a water bath at 30 °C for 10 seconds (Fig. 1).

#### Sperm function

The following sperm quality parameters were evaluated in both fresh and thawed semen: sperm motility (%), spermatozoa movement duration (s), viability (%) and DNA integrity (%). Sperm concentration in fresh semen was also measured by a Neubauer chamber. The semen was diluted 1/1000 (v:v) with 3% NaCl (w:v) and sperm counts were performed in duplicate at a magnification of 400× and expressed as ×10^9^/mL.

The sperm motility of fresh semen was subjectively evaluated by placing an aliquot of semen (1 μL) on a glass microscope slide with 10 μL of an activation solution (0.3% NaCl). Observation was carried out at room temperature (15–20 °C) and sperm motility was expressed as the percentage of motile spermatozoa observed under 40× magnification. Sperm were defined as motile if they showed forward movements, whereas simply vibrating sperm were deemed immobile.

The motility parameters of cryopreserved semen were examined using a computer-aided sperm analysis system coupled to a phase contrast microscope (Nikon Eclipse model 50i; negative contrast) employing the Sperm Class Analyzer (SCA) software (version 4.0, Microptic S.L., Barcelona, Spain) with the Makler counting chamber (Sefi Medical Instruments, Haifa, Israel). Sperm were activated at a dilution of 1:10 with 1% NaHCO_3_.

The following sperm motility parameters were evaluated: motile spermatozoa [MOT, (%)], curvilinear velocity [VCL, (µm/s)], straight-line velocity [VSL, (µm/s)], average path velocity [VAP, (µm/s)], amplitude of lateral head displacement [ALH, (µm)], beat cross frequency [BCF, (Hz)], linearity [LIN, (%)], straightness [STR, (%)] and wobble [WOB, (%)].

The duration of sperm movement (DSM) was measured using a sensitive chronometer as soon as the activation solution was added.

The integrity of plasma membrane was assessed using the LIVE/DEAD Sperm Viability Kit (Molecular Probes, Inc.) containing the fluorescent stains SYBR-14 and propidium iodide (PI). This procedure was performed on 1 μL of fresh or thawed semen, which were added to 40 μL of an immobilizing medium (80 mM NaCl, 40 mM KCl, 0.1 Mm CaCl_2_, 30 mM Tris-HCl, pH 9.2) (v/v). A total of 2.5 µL of SYBR-14 working solution (50-fold dilution in distilled water of the stock solution-10-fold dilution in DMSO of the SYBR-14 commercial solution) were added to the cell suspension. After 10 minutes of incubation at room temperature in the dark, 3 µL of working PI solution (PI solution diluted 1:100 in phosphate buffered saline (PBS) diluent were added to the cell suspension. The spermatozoa were incubated for an additional 10 minutes under the same conditions. Ten microliters of this suspension was then placed on microscope slides, covered with coverslips and examined at a magnification ×1000 using a ×100 oil immersion objective under epifluorescence illumination. For each sample, approximately 200 spermatozoa were examined in duplicate aliquots. SYBR-14 is a membrane-permeant DNA stain, which only stains live spermatozoa producing a green fluorescence of the nuclei, while propidium iodide stains the nuclei of the membrane-damaged cells in red. Thus, spermatozoa showing green fluorescence are scored as alive and those showing red fluorescence as dead. The percentage of viable spermatozoa was calculated as the number of green cells ×100 divided by the total number of sperm counted.

Sperm DNA integrity was evaluated using acridine orange (AO) following the method described by Gandini *et al*.^34^. We adapted this test following the procedure used for rabbit semen in our previous paper^35,36^. AO is a cationic fluorescent cytochemical that stains cell nuclei, specifically DNA. Acridine orange fluoresces green when incorporated into native DNA (double-stranded and normal) as a monomer, and orange-red when it binds to denatured (single-stranded) DNA as an aggregate.

This test was performed on 1 μL of fresh or thawed semen, which was added to 40 μL of immobilizing medium (80 mM NaCl, 40 mM KCl, 0.1 mM CaCl_2_, 30 mM Tris-HCl, pH 9.2) (v/v). Then, 10 µL of this solution was smeared onto a microscope slide, air-dried and fixed overnight in a 3:1 methanol:glacial acetic acid solution, and air-dried once more. The slides were washed with distilled water and the smears were stained with an AO solution (0.2 mg/mL in water) for 5 minutes in the dark at room temperature. Each smear was then washed with distilled water, mounted with a coverslip and examined using a fluorescence microscope with a 490 nm excitation light and a 530 nm barrier filter. Nuclei in at least 200 spermatozoa per slide were examined and scored as fluorescing green or yellow-orange-red (intact DNA or damaged DNA respectively) and the percentage of normal and abnormal chromatin condensation was also calculated.

### Experiment 2. *In vivo* reproductive capacity of semen cryopreserved using the most effective P-CPA identified for each extender in Experiment 1

**Figure 2:**
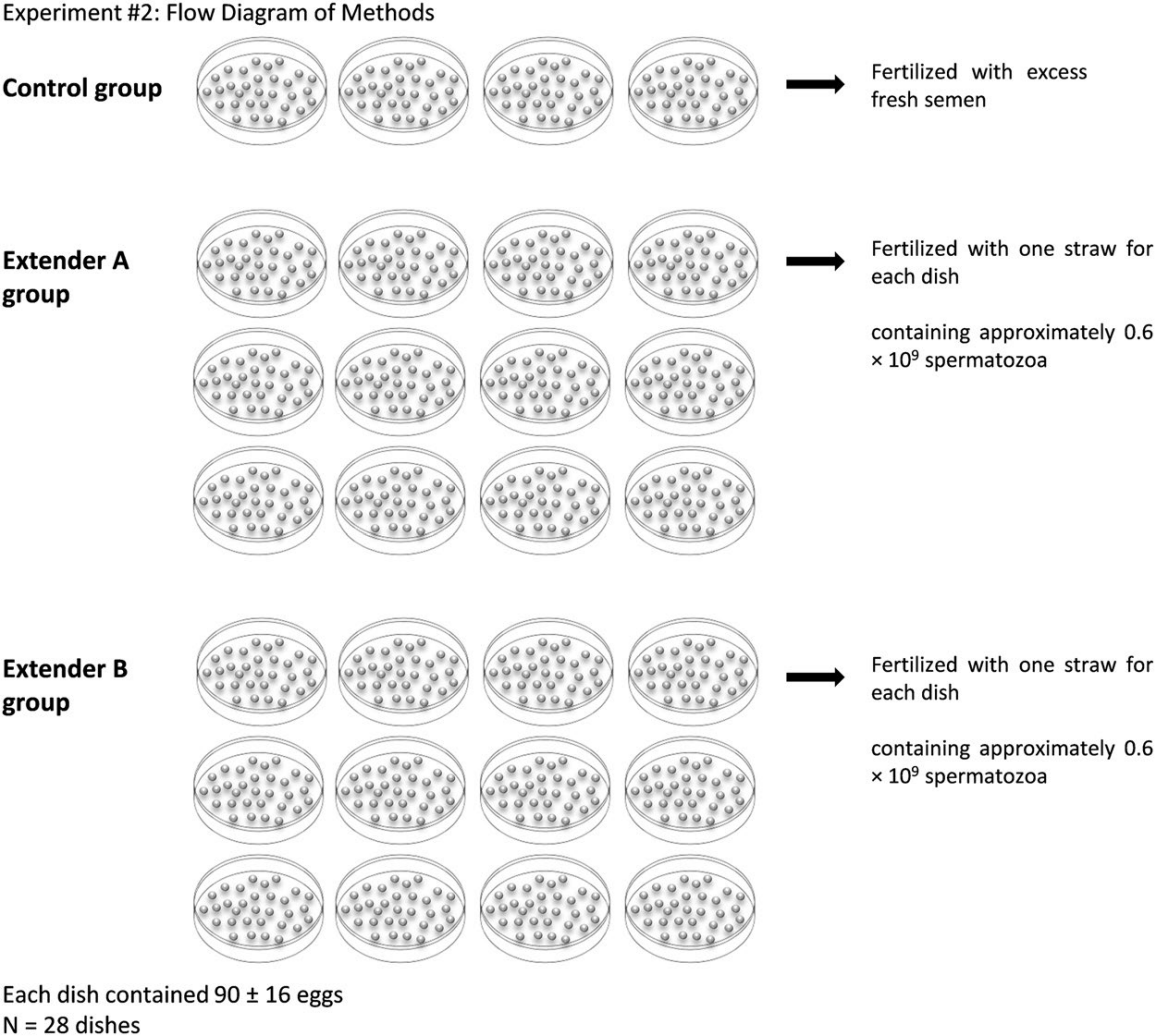
Flow diagram of the *in vivo* experiment (Experiment 2) to assess the reproductive capacity of cryopreserved semen.

Based on the results obtained in (1), we compared *in vivo* semen cryopreserved using the most effective P-CPA identified (DMSO) for each extender (A and B) with fresh semen in an artificial fertilization trial. Fertilization was performed using 28 dry plastic dishes, and three treatment groups were created: (1) 4 dishes fertilized with fresh semen (control group); (2) 12 dishes fertilized with cryopreserved semen using extender A containing DMSO (group A); and (3) 12 dishes fertilized with semen frozen using extender B combined with DMSO (group B) (Fig. 2). Eggs were collected from seven females and mixed together. A total of 90 ± 16 eggs were placed on each dish, and 5 ml of D532 (20 mM Tris, 30 mM glycine, 125 mM NaCl, pH 9.0)^37^ as a fertilization solution was subsequently added to the eggs in each of 28 dishes. The sperm was immediately added and the gametes were gently mixed for 10 seconds. For the control group, excess fresh semen was used at the beginning and end of the fertilization trials to test egg quality, while for the frozen treatment groups A and B, 0.25 mL (one straw containing approximately 0.6 × 10^9^ spermatozoa) of thawed semen (30 °C for 10 seconds) was used for each dish. Then, approximately 20 mL of hatchery water was added to the control and frozen treatment groups. After 2 minutes, the eggs were rinsed with hatchery water and transferred to perforated baskets (diameter 6 cm), incubated in a longitudinal hatchery tank with running water at temperature of 9 °C. Unfertilized and dead eggs were continuously counted and removed. After 25–30 days, the eggs had reached the eyed-egg stage, and 45–50 days after fertilization, the embryos started to hatch. The fertilization success rate was established by calculating the percentage of embryos at both the eyed- and hatching-larvae stages. We calculated the percentage of eyed embryos and hatching larvae using the initial number of eggs calculated as the number of eyed eggs or hatchings larvae × initial egg number^−1^ × 100.

### Statistical analysis

To compare the different treatments, we used a randomized block design in a 2 × 3 factorial arrangement (2 extenders × 3 P-CPAs), with 12 replicates per treatment. A generalized linear model (GLM) procedure was used to determine the fixed effects of the extender, the type of P-CPA and the effects of their interaction on the sperm quality variables. Sperm variables (motility percentage and duration, viability and DNA integrity) and fertilization and hatching measured among the different treatments were compared by ANOVA (analysis of variance) followed by Scheffe’s comparison test. Significance was set at P < 0.05. All statistical tests were performed using the software package SPSS (SPSS 15.0 for Windows, 2006; SPSS, Chicago, Ill).

### Ethics approval and consent to participate

The experiments were carried out in accordance with the guidelines of the current European Directive (2010/63/EU) on the care and protection of animals used for scientific purposes.

## Results

### Effects of different extenders and P-CPAs on post-thaw semen quality

Spermatozoa motility (%) and their duration (seconds) in fresh semen were 81.2 ± 5.7 and 46.3 ± 6.1, sperm viability and DNA integrity (%) were 83.8 ± 3.3 and 97.6 ± 1.2 respectively, and the average sperm concentration was 10.6 ± 1.4 × 10^9^ sperm/mL.

The fixed effects of the extender and type of P-CPA combination on sperm viability and DNA integrity are shown in Table 1. The data obtained indicate a significant effect of the extender and the P-CPA (P < 0.05) on both of these parameters, while no significant interaction effect was observed between the extender and the P-CPA.

**Table 1:** Sperm viability and DNA integrity (mean ± SE) recorded for semen of trout from the Biferno River frozen with different extenders and P-CPAs (N = 12).

Semen treatment		Sperm variable	
Extender	P-CPA	Viability (%)	DNA integrity (%)
A	DMSO	36.3 ± 1.1^a^	97.3 ± 1.2^a^
A	MeOH	22.3 ± 3.1^b^	86.9 ± 0.4^c^
A	DMA	22.1 ± 1.6^b^	91.5 ± 0.8^b^
B	DMSO	29.9 ± 1.9^a^	97.1 ± 0.3^a^
B	MeOH	13.3 ± 1.9^c^	83.5 ± 0.9^d^
B	DMA	17.9 ± 3.2^bc^	89.9 ± 0.5^b^
Extender effect P-CPA effect Extender × P-CPA effect		P < 0.001	P < 0.001
		P < 0.000	P < 0.000
		P < 0.543	P < 0.082

^a–d^Different superscript letters within the same column indicate a significant difference (P < 0.05).

Extender: A (glucose 300 mM); B (NaCl 75 mM, KCl 70 mM, CaCl_2_ 2 mM, MgSO_4_ 1 mM, TRIS 20 mM)

P-CPA: penetrating cryoprotectant; DMA: dimethylacetamide; DMSO: dimethylsulfoxide; MeOH: methanol

Higher values of viability and DNA integrity were recorded in semen frozen in the presence of DMSO both in extender A and extender B (P < 0.05). Lower viability and DNA integrity values were found for extender B containing MeOH.

The motility parameters recorded in frozen/thawed semen are provided in Table 2. These data indicate a significant effect of the extender used (P < 0.05) on total motility, VCL, VSL, VAP, LIN and DSM. The type of P-CPA significantly affected total motility, VSL, VAP, LIN, STR, WOB and DSM, while the interaction of the extender*P-CPA affected the total motility, VCL, VSL, VAP, ALH and BCF (P < 0.05).

**Table 2:** CASA parameters and duration of sperm movement (mean ± SE) recorded for semen of trout of the Biferno River frozen in the presence of different extenders and P-CPAs (N = 12).

Semen treatment		Sperm motility parameters									
Extender	P-CPA	Total Motility (%)	VCL (µm/s)	VSL (µm/s)	VAP (µm/s)	LIN (%)	STR (%)	WOB (%)	ALH (µm)	BCF (Hz)	DSM (sec)
A	DMSO	42.6 ± 3.5^a^	22.8 ± 0.5^a^	10.2 ± 0.3^a^	16.1 ± 0.4^a^	44.7 ± 0.9^a^	62.9 ± 0.6^a^	70.9 ± 1.1^a^	3.5 ± 0.1^a^	2.3 ± 0.3^a^	40.7 ± 2.1^a^
A	MeOH	24.6 ± 2.9^b^	19.2 ± 0.6^b^	7.8 ± 4.5^b^	13.1 ± 0.6^b^	40.3 ± 1.6^ab^	59.2 ± 1.1^ab^	67.8 ± 1.5^ab^	2.6 ± 0.4^ab^	2.2 ± 0.5^a^	29.3 ± 1.2^b^
A	DMA	14.3 ± 2.4^c^	18.9 ± 0.8^b^	7.1 ± 0.6^bc^	12.2 ± 0.9^b^	36.9 ± 2.4^bc^	58.1 ± 1.2^b^	63.5 ± 3.6^bc^	1.9 ± 0.6^bc^	0.9 ± 0.3^bc^	24.8 ± 1.7^bc^
B	DMSO	20.9 ± 2.1^bc^	19.7 ± 0.6^b^	7.2 ± 0.3^bc^	12.6 ± 0.5^b^	42.9 ± 3.4^a^	61.7 ± 2.9^a^	67.2 ± 3.6^ab^	0.9 ± 0.5^c^	0.5 ± 0.2^c^	30.6 ± 0.7^b^
B	MeOH	18.4 ± 1.3^bc^	16.5 ± 0.9^c^	7.0 ± 0.5^bc^	11.2 ± 0.9^b^	36.6 ± 0.9^bc^	56.9 ± 0.9^b^	64.2 ± 1.3^bc^	2.5 ± 0.5^ab^	1.4 ± 0.5^bc^	20.7 ± 2.2^c^
B	DMA	17.5 ± 1.5^bc^	19.5 ± 0.5^b^	6.4 ± 0.3^c^	11.6 ± 0.4^b^	32.7 ± 1.4^c^	54.7 ± 1.2^b^	59.6 ± 1.7^c^	2.8 ± 0.4^ab^	1.7 ± 0.4^ab^	21.4 ± 2.7^c^
Extender effect		P < 0.000	P < 0.002	P < 0.000	P < 0.000	P < 0.047	P < 0.196	P < 0.061	P < 0.088	P < 0.069	P < 0.000
P-CPA effect		P < 0.000	P < 0.817	P < 0.000	P < 0.029	P < 0.000	P < 0.000	P < 0.009	P < 0.749	P < 0.399	P < 0.000
Extender × P-CPA effect		P < 0.000	P < 0.000	P < 0.006	P < 0.001	P < 0.841	P < 0.386	P < 0.999	P < 0.001	P < 0.004	P < 0.196

^a–c^Different superscript letters within the same column indicate a significant difference (P < 0.05).

Extender: A (glucose 300 mM); B (NaCl 75 mM, KCl 70 mM, CaCl_2_ 2 mM, MgSO_4_ 1 mM, TRIS 20 mM).

P-CPA: cryoprotectant; DMA: dimethylacetamide; DMSO: dimethylsulfoxide; MeOH: methanol.

Total motility: the percentage of motile spermatozoa; VCL: curvilinear velocity; VSL: straight-line velocity; VAP: average path velocity; ALH: amplitude of lateral head displacement; BCF: beat cross frequency; LIN: linearity (VSL/VCL × 100); STR: straightness (VSL/VAP × 100), WOB: wobble (VAP/VCL × 100) and DSM: duration of sperm movement.

The best post-thaw total motility, VCL, VSL, VAP and DSM were recorded for semen cryopreserved with extender A/DMSO (P < 0.05).

For both extenders the lower values of total motility, VCL, VSL, LIN, STR and WOB were recorded in semen frozen/thawed in the presence of DMA.

Based on these findings, extenders A and B combined with DMSO were used in the *in vivo* artificial fertilization trial as the most effective treatments.

### Fertilization ability of cryopreserved semen

The percentage of eyed eggs and hatching rates recorded for cryopreserved and fresh semen are provided in Table 3.

**Table 3:** Fertilization ability of fresh semen or semen frozen in the presence of extender A or B combined with DMSO.

	Semen treatment		
	Fresh	Frozen	
		Extender A	Extender B
Eyed eggs (%)	83.7 ± 1.2^a^	36.5 ± 5.5^b^	27.8 ± 4.2^b^
Hatching rate (%)	75.5 ± 1.6^a^	32.5 ± 4.9^b^	23.1 ± 3.5^b^

Values with different superscript letters within treatments of the same row are significantly different (P < 0.05).

Extender: A (glucose 300 mM); B (NaCl 75 mM, KCl 70 mM, CaCl_2_ 2 mM, MgSO_4_ 1 mM, TRIS 20 mM); DMSO: dimethylsulfoxide.

The percentage of eyed and hatched eggs was significantly higher in fresh semen compared to frozen semen. No significant differences emerged when we compared the frozen semen using extender A or B, although, the higher percentages of eyed eggs and hatching rates were recorded in eggs fertilized with extender A.

## Discussion

Obtaining effective semen cryopreservation protocols is an important goal because sperm cryopreservation has several advantages for biodiversity conservation, such as minimizing inbreeding and reducing domestication selection. Fish semen cryopreservation is currently the only technology available to develop *ex situ* conservation programmes because oocyte and embryos cryopreservation remain unsatisfactory. Importantly, sperm cryopreservation techniques have been developed for a wide variety of endangered salmonids^2^ but seldom used for *S. macrostigma*.

We recently studied the optimal freezing rate for the semen cryopreservation procedure for the Mediterranean brown trout of the Biferno River^22^. The freezing rate is only one of the steps of the semen cryopreservation procedure, and further improvements of other steps of the freezing protocol are necessary. This is even more important considering that sperm cryotolerance could vary among native trout populations.

This study sought to identify the most effective basic extender and P-CPA as well as the best combination between the type of extender and P-CPA for semen cryopreservation in wild specimens of the Mediterranean brown trout (*Salmo cettii*) population of the Molise river (Italy). In this regard, some researchers have indeed shown that the effectiveness of sperm cryopreservation may also depend on the interaction between the type of extender and cryoprotectant employed^25,38^.

### Effects of different extenders and P-CPAs on *in vitro* post-thaw semen quality

Our *in vitro* results clearly revealed a significant effect of the extender and P-CPA on the quality of the cryopreserved semen. In fact, extender A combined with DMSO produced an overall high post-thaw semen quality compared with all the other combinations. Likewise, extender B combined with DMSO showed higher post-thawing quality compared with MeOH and DMA.

As reported in the literature, the choice of a suitable extender and P-CPA is key to successful cryopreservation of salmonid sperm^23^.

Here, the choice of the extenders was addressed by previous research that reported a better post thawing *in vitro* semen quality with a mineral-based extender^32^ and glucose-based extender^31^. Our results also revealed that the glucose-based extender (extender A) provided better *in vitro* conditions than the mineral-based extender (extender B) to preserve sperm integrity (viability and DNA) and function (motility) during the freezing/thawing procedure. This is consistent with the finding of Bozkurt and Yavas^23^ reporting that carbohydrate-based extenders are preferred with respect to other extenders. These authors established that the glucose-based extender provided higher post-thaw motility and duration with respect to the mineral extender (Lahnsteiner extender). Specifically, glucose-based extenders have been used for the cryopreservation of rainbow trout^31^ and brown trout sperm^23,25^ with satisfactory results. Hence, the success of extender A could be explained by the ability of glucose to protect the sperm from osmolality damage as reported by Leung and Jamieson^39^; Maisse^40^ also showed that the efficacy of sugars as extenders can be explained by their role as external CPA and membrane stabilizers. In this regard, various authors have recently shown the positive effects of glucose and trehalose as external CPAs in semen cryopreservation protocols of rainbow and brown trouts^33,41–43^.

Another interesting point emerging from this research is that DMSO provided better post-thaw sperm quality than DMA and MeOH for both tested extenders. The CPA molecules used here are classified as permeable CPAs, and their mechanism of action is the same; therefore, the reason why DMSO provided better results is the object of our speculation. P-CPAs are membrane-permeable solutes that act intra- and extracellularly, causing the dehydration of spermatozoa because of an osmotically driven flow of water, which varies according to CPA composition^44–46^. Spermatozoa are normally equilibrated in a P-CPA, preventing cells from undergoing intracellular ice-crystal formation, which is mainly responsible for cell damage affecting the plasma membrane, mitochondria and chromatin structure^47^.

Penetrating CPAs also cause membrane lipid and protein reorganization. This improves membrane fluidity, causing greater dehydration at lower temperatures, and thus an increased ability to survive cryopreservation^48^. In light of these considerations, DMSO was associated with less physical-functional injuries to the sperm and was better than DMA and MeOH at preserving the post-thaw semen quality of *Salmo cettii* under our experimental conditions. This leads us to hypothesize that although the P-CPA molecules act in the same way, they have different chemical-physical properties, specifically in terms of molecular weight (DMSO 78.13, DMA 87.12 and methanol 32.04 gmol-1) and chemical functional groups (DMA-amide groups, DMSO-hydrophilic sulfoxide group and MeOH-alcoholic group). These properties are likely to confer upon the compounds a different degree of permeability in a given phospholipid bilayer and lesser or greater cellular toxicity. In turn, this might lead to variations in the relative cryoprotection efficiency of these CPAs for *S. cettii* sperm. Our results depict a clear scenario in which, because of its lower molecular weight compared to other cryoprotectants (DMSO and DMA), MeOH is highly permeable to cell membranes but, on the other hand, is also more toxic^49^, while DMSO is more permeable to sperm membranes than DMA and less toxic than the other compounds. In this regard, this notion is also substantiated by Noble^49^, who reported DMSO to be the most widely used P-CPA because it showed the right compromise between its membrane permeability and toxicity.

A variety of P-CPAs, such as DMSO, DMA, MeOH, glycerol and ethylene glycol, have been tested for the cryopreservation of brown trout^11,13,14,23,25,50^ and rainbow trout semen^10,15,41,51^. In general, DMSO and MeOH at different concentrations were mainly used as P-CPAs in freezing protocols for trout semen, whereas little is known about the usage of DMA. In particular, Ciereszko *et al*.^41,52^ and Dietrich *et al*.^53^ reported that MeOH is a suitable P-CPA for sperm cryopreservation of rainbow trout, while DMSO is widely used for brown trout^23^. However, in some papers, MeOH has also been successfully used in brown trout cryopreservation^13,20^.

### Fertilization capacity of cryopreserved semen

Interestingly, our *in vivo* study reported no significant differences in the number of eyed eggs and hatching rates between the considered extenders, although these fertilization parameters were notably higher (approximately 10% more) for extender A. The fertilization ability found here was higher than that observed in our previous paper^22^ due to the different sperm-to-egg ratios applied. In this regard, we used a ratio of approximately 6 × 10^6^ sperm/egg, while in our previous research, a ratio approximately 10 times lower (0.5 × 10^6^ sperm/egg) was applied.

Therefore, we think that the sperm-to-egg ratio used here is more appropriate for artificial fertilization in the Mediterranean brown trout of the Biferno River as a result of a higher concentration of viable and motile spermatozoa suitable for each egg. This aspect emphasizes the importance of the choice of the optimal spermatozoa/egg ratio to determine the fertilization capacity of thawed sperm, as reported by different authors^20,23,43^. In addition, the sperm-to-egg ratio used here was similar to that reported in brown trout by Sarvi *et al*.^50^ with 6.2 × 10^6^ sperm/egg, while 4 × 10^6^ sperm/egg was chosen by Dziewulska and Domagała^20^.

*In vivo* results were similar to those recorded in brown trout by Labbé and Maisse^16^ and Bozkurt and Yavas^23^ and were lower than those reported by other authors^13,20,50,54^.

The literature reports how the variability in the biological material and the use of multiple preservation procedures have made it impossible to reproduce either the quality or the fertilizing capacity of cryopreserved semen^12,14^. In addition, susceptibility to semen cryopreservation varies among fish species^12^, within subpopulations^14^. This notion is substantiated by Martínez-Páramo *et al*.^14^, who observed different fertilization rates using cryopreserved semen from two brown trout subpopulations inhabiting different rivers in the same basin. This means that the sperm of different populations belonging to the same fish species have different biological characteristics and consequently dissimilar cryoresilience, so an individualized semen cryopreservation protocol is also required.

Thus, the present study showed that an extender composed of 300 mM glucose combined with 10% DMSO and 10% egg yolk resulted in remarkably high post-thaw quality *in vitro* and in a better fertilizing ability in semen of the Mediterranean brown trout of the Molise rivers. Therefore, the achievement of an effective semen cryopreservation protocol for *S. cettii* will contribute to the creation of a sperm cryobank that is an important tool for the conservation of the biodiversity of this Molise native trout.

## Conclusions

Our results identified the glucose-based extender and DMSO as the best combination for an effective cryopreservation protocol for the native trout of the Molise rivers. However, further studies are needed to improve the semen freezing protocols for this trout by studying the NP-CPA, equilibration time, thawing rate and cryopreserved sperm-to-egg ratio.

Our findings are important because they will allow the creation of a sperm cryobank that is key to the conservation and restoration of the native population of the Mediterranean brown trout (*Salmo cettii*) in Molise rivers. The use of cryopreserved semen in artificial fertilization protocols represents a valuable tool to maintain genetic diversity and fitness within self-sustaining populations. Furthermore, the creation of the first sperm cryobank of pure *Salmo cettii* populations with high genetic variability will be useful not only for Molise river basins but also for other Italian basins where this species is at risk of extinction.

## Data Availability

The datasets used and/or analysed during the current study are available from the corresponding author upon reasonable request.
